# Disparities in Chronic Stress Exposure and Appraisal and Later-Life Disability

**DOI:** 10.1093/geroni/igaf024

**Published:** 2025-04-04

**Authors:** Madison R Sauerteig-Rolston

**Affiliations:** Department of General Internal Medicine and Geriatrics, Indiana University School of Medicine, Indianapolis, Indiana, USA; Regenstrief Institute, Indiana University Center for Aging Research, Indianapolis, Indiana, USA

**Keywords:** Chronic stressors, Ethnicity, Nativity, Negative appraisal, Race

## Abstract

**Background and Objectives:**

Influenced by the stress process theory, this study investigated the relationship between chronic stress (measured by exposure and appraisal) and the onset of a disability in later life among White, Black, U.S.-born Hispanic, and foreign-born Hispanic adults.

**Research Design and Methods:**

Using nationally representative data from the Health and Retirement Study, I used Weibull accelerated failure time models to examine racial, ethnic, and nativity disparities in chronic stress exposure and appraisal and age of onset of disability during the following 8–10 years (i.e., incidence).

**Results:**

Over time, earlier onset of disability was associated with higher levels of stress exposure (β = −0.04) and negative appraisals (β = −0.07). Appraising stress as more upsetting had a detrimental influence on later-life disability for Black adults (occurring 11% earlier), but a protective effect for foreign-born Hispanic adults (occurring 20% later) compared with White adults.

**Discussion and Implications:**

Overall, findings suggest it is important to acknowledge not just the exposure to chronic stressors, but how upsetting these chronic stressors make one feel to reduce racial, ethnic, and nativity disparities in disability.

**Translational Significance:** Understanding the subjective dimensions of stress may provide a more comprehensive understanding of disparities in the association between stress and health, particularly disability. Indeed, appraising chronic stress as more upsetting has a detrimental influence on later-life disability, especially among Black adults. In contrast, I find higher levels of negative appraisal to exude a protective effect for foreign-born Hispanic adults. To reduce disparities in the onset and burden of disability, interventions should aim to alleviate the negative emotions that come with chronic disadvantages and find constructive ways to cope with stressors judged to be upsetting.

A large body of literature reveals that stress exposure is unequally distributed across the U.S. population, with Black and Hispanic people more likely to experience negative events throughout the life course (Boen & Hummer, 2019; Ferraro et al., 2017; Phelan & Link, 2015; Sauerteig-Rolston & Ferraro, 2024; Sternthal et al., 2011). Stress process theory speculates that this disproportionate exposure to stress is in part a plausible explanation for racial and ethnic health disparities (Turner, 2009). Despite the importance of these prior studies, stress exposure is only one dimension of the stress process. An understudied but critical component of how stress may have long lasting effects on health is stress appraisal, the extent to which individuals internalize and interpret a stressor to be upsetting. Experiencing the same event can be upsetting for some individuals, but not for others, depending on how one evaluates the exposure (Brown et al., 2020a; Kessler, 1979).

The motivation for this study was sparked by Brown and colleagues (2020a) study in which they found that despite experiencing a greater number of chronic stressors, Black and U.S. born Hispanic adults were less likely to be upset by the exposure compared with White adults. Brown and colleagues (2020a) emphasized: “Individuals do not experience stress in a vacuum, but rather in the context of different personal and environmental resources that shape the stressfulness of a life experience” (p. 652). It is intriguing that exposure may be appraised as less upsetting by marginalized groups that often have fewer resources to cope with or mitigate negative experiences; further exploration is warranted to understand how stress appraisal influences racial and ethnic health disparities.

One health disparity in later life that has implications for independence and quality of life is activities of daily living (ADLs) disability (hereafter, disability). ADLs are composed of basic, necessary, socially defined tasks, such as bathing and dressing (Verbrugge & Jette, 1994). Many studies have revealed that Black and Hispanic adults have a higher prevalence of disability compared with White adults (e.g., Heimbuch et al., 2023; Shipeolu et al., 2024; Vásquez et al., 2020), and foreign-born Hispanic adults report living more years of life with a disability than White, Black, and U.S.-born Hispanic adults (Hayward et al., 2014). Understanding the mechanisms that influence disparities in the age of disability onset may improve the health span of adults by providing them with more years of being able to function independently.

A couple studies have examined racial, ethnic, and nativity disparities in the association between stress exposure and disability, yet more research is warranted that examines stress *appraisal* as a potential factor that may alter the onset of a disability. This study uses longitudinal data from a diverse national sample to examine racial, ethnic, and nativity disparities in chronic stress exposure and appraisal and the transition to a disability in later life. I focus on several domains of chronic stress across multiple facets of life (i.e., multi-domain chronic burden), including financial strain, relationship problems, and health problems, to determine how these enduring stressors are associated with a disability among White, Black, U.S.-born Hispanic, and foreign-born Hispanic adults.

## The Stress Process and Chronic Stress Exposure

The stress process model posits that stress arises out of two broad circumstances: the occurrence of discrete (event-based) stressors or the presence of life strains (chronic stressors; Pearlin et al., 1981). Chronic stressors are ongoing strains in one’s social environment that are often persistent, recurrent, and interconnected—and may cause wear and tear on one’s body systems over time (Pearlin et al., 1981; Thoits, 2010). Chronic stressors are argued to exert a heavy impact because they often occur in roles that have high importance for individuals and society (Pearlin et al., 2005). Indeed, these strains tend to emerge in major life domains such as family relationships, financial stability, and health with no easy solution and often require ongoing attention and coping (Brown et al., 2020a; Pearlin et al., 2005). Event-based stressors and chronic stressors have distinct characteristics; however, Pearlin and colleagues (1981) contended that event-based stressors may create or intensify life strains.

Particularly important for this study, the combination of chronic stressors across several domains of life may be more influential for negative health outcomes than one chronic stressor. Multi-domain chronic burden (hereafter, chronic stress exposure) represents the cumulative value of stress exposure across multiple domains of life stress (Lockwood et al., 2022). Studies reveal that multi-domain chronic burden is associated with many adverse health outcomes including higher levels of depressive symptoms (Brown et al., 2020b), chronic pain (Spector et al., 2023), and insomnia symptoms (Kuo et al., 2023), as well as increased risk of myocardial infarction (Dupre et al., 2021).

To the best of my knowledge, however, it is not known how chronic stress exposure may influence racial, ethnic, and nativity inequalities in later-life disability. Several studies have reported an association between cumulative stress exposure across the life course and disability (Leger et al., 2018; Morton, 2022; Shrira & Litwin, 2014). Yet, few studies have examined racial, ethnic, and nativity disparities in the association between stress burden and physical disability (Bowen, 2009; Sauerteig-Rolston & Ferraro, 2024). For example, Sauerteig-Rolston and Ferraro (2024) found that a higher level of cumulative stress burden, measured as the sum of affirmative responses to 35 stressors (assessed in childhood and adulthood) was associated with an earlier onset of disability in later life, especially for U.S.-born Hispanic adults. Many stressors in this study were event-based stressors, such as: “Were you ever physically abused as a child?” and “Have you ever been stopped by the police?” Although these discrete, potentially traumatic, and often sudden events have long lasting influences on many health outcomes in later life, it is equally as important to understand and disentangle the influence of *enduring* chronic stressors, those that may represent the presence of continuous problems.

## Disparities in Multi-Domain Chronic Stress Appraisal and Health

Appraising a stressor as upsetting, perhaps more than just the presence of a stressor, is what activates a body’s stress response system (Lazarus & Folkman, 1984; Morris et al., 2022). Stress appraisal is the reaction to a stressful experience that “gets under the skin” to influence health above and beyond the exposure. For example, some research on family caregiving portrays it as a stressful and burdensome experience with negative health outcomes for the caretaker, yet others report positive health effects of family caregiving (Roth et al., 2015). As such, I argue that it is more important to evaluate the emotional reactivity to stress, rather than the exposure. Several studies have examined the association between emotional reactivity to stress and disability (Leger et al., 2018; Rush et al., 2025). For example, Leger and colleagues (2018) examined the lingering influence of negative affect (e.g., irritable, upset, etc.) after a daily stressor (e.g., having something bad happen to a close friend) and found that increases in negative affect were associated with more disability in later life.

Relatively few studies examine how race, ethnicity, and nativity may moderate the relationship between chronic stress appraisal and health. Those that do examine Black-White differences in either mental health outcomes (Brown et al., 2020b) or cognitive health outcomes (Morris et al., 2022). For example, Brown et al. (2020b) found that Black adults who reported greater exposure to chronic stressors reported fewer depressive symptoms than White adults with a similar level of chronic stress. After accounting for stress appraisal, however, the interaction between race and exposure was no longer significant, revealing that whether a stressor is considered upsetting may be one mechanism by which Black older adults reduce the detrimental effects of stress exposure on mental health (Brown et al., 2020b).

It remains unclear what influence chronic stress appraisal has on physical health outcomes and if the protective effect found for Black adults in terms of mental and cognitive health (a) extends to physical health and (b) is consistent for other minority groups. Stress appraisal may provide important insight into racial, ethnic, and nativity differences in disability.

## The Current Study and Research Questions

This study aims to (a) identify racial, ethnic, and nativity disparities in chronic stress exposure, appraisal, and incident disability, (b) utilize event history analysis to examine the relationship between chronic stress exposure, appraisal, and the age of onset of first disability, and (c) test if the relationships between exposure, appraisal, and the onset of disability varies by race, ethnicity, and nativity. As such, the present study is guided by the following research questions:

1. *Do levels of chronic stress exposure, chronic stress appraisal, and disability differ by race, ethnicity, and nativity?*
2. *Do exposure and appraisal to chronic stressors influence the age of onset of disability?*
3. *Does the relationship between chronic stress exposure and appraisal on risk of disability onset vary by race, ethnicity, and nativity?*

## Research Design and Methods

### Sample

This study used longitudinal data from the Health and Retirement Study (HRS), a nationally representative, biennial panel study of American adults aged 50 years and older. Due to the survey design, respondents in the HRS were randomly selected to answer questions for the chronic stress variables in either the 2010 or 2012 psychosocial questionnaire. To optimize the use of variables from this half-sample questionnaire, wave 1 (W1) disability and most covariates were gathered in either 2010 or 2012, matching participation in the psychosocial questionnaire, and respondents were followed prospectively through 2020 (W8). More information about the design of the HRS is presented by Sonnega et al. (2014).

The analysis sample was limited to respondents who met the following criteria: (a) participated and had nonzero weights (i.e., 50 + years of age, noninstitutionalized) in the 2010 or 2012 wave including the psychosocial module (*N* = 14,664), (b) reported no disability prior to or at the 2010 or 2012 data collection, depending on one’s psychosocial questionnaire (*N* = 10,609), (c) self-identified as U.S.-born non-Hispanic White, U.S.-born non-Hispanic Black, U.S.-born Hispanic, or foreign-born Hispanic (*N* = 9,833), (d) scored greater than six on cognition at W1 (≤6 indicates presence of dementia on the modified version of the TICS survey) (*N* = 9,644), (e) provided responses to at least one of the chronic stressor measures (*N* = 9,474), and (f) were not missing on any covariates in the study (*N* = 9,171). Finally, respondents who did not experience any of the stressors were excluded from the models that incorporated stress appraisal (1,491 respondents excluded; *N* = 7,680).

### Measures

#### Disability

The focal dependent variable in this study was first onset of an *ADL disability* during the study period. HRS respondents were asked whether they had any difficulty (a) bathing, (b) eating, (c) dressing, (d) walking across a room, or (e) getting in or out of bed. This study utilized the summary score created by RAND, which combined all ADL limitations into one count variable, ranging from 0-5 (Bugliari et al., 2023). Because I was interested in the onset of first disability, I recoded the count measure into a binary variable, where 1 indicated at least one disability.

#### Chronic stress exposure and appraisal

Chronic stress was conceptualized by two domains: exposure and appraisal. Respondents reported whether they had experienced any of the following seven ongoing problems for 12 months or longer during the 2010 or 2012 psychosocial questionnaire: health problem (in yourself), physical or emotional problem (in spouse or child), problems with alcohol or drug use in family member, financial strain, housing problems, problems in a close relationship, and/or helping at least one sick/limited/or frail family member or friend on a regular basis. Response options for each stressor included the following: “no, didn’t happen” (coded as 0), “yes, but not upsetting” (1), “yes, somewhat upsetting” (2), and “yes, very upsetting” (3).

To calculate the *chronic stress exposure* variable, a sum measure was created indicating whether the respondent endorsed each of the seven stressors, ranging from 0 to 7 (α = 0.61). Then, following the lead of others (Brown et al., 2020a; Morris et al., 2022), the *chronic stress appraisal* variable was calculated by averaging responses across all stressors that were endorsed, ranging from 1 to 3 (α = 0.77). For example, if a respondent reported experiencing five out of the seven stressors, their appraisal score represented the average of those five stressors. Taking the average appraisal score ensured that stress appraisal scores were not dependent on stress exposure scores, but these measures were correlated (*r* = 0.19).

#### Race, ethnicity, and nativity

Four binary variables (0,1) were used to assess *race, ethnicity, and nativity*: White (reference group), Black, U.S.-born Hispanic, and foreign-born Hispanic. This study accounted for nativity status among Hispanic adults only due to the low percentage of White and Black adults in the HRS born outside of the United States. Given their low frequency in the HRS, foreign-born White (*n* = 319) and foreign-born Black (*n* = 101; after all other exclusion criteria) respondents were excluded from this study to ensure results were not affected by combining nativity status within the White and Black racial subgroups.

#### Covariates

This study adjusted for characteristics known to influence disability in later life. A binary variable was utilized to differentiate *men and women* (0 and 1, respectively). Three adult resources were adjusted for: *marital status* (married vs nonmarried), *education* (years schooling, top coded at 17+) and *household wealth* (assets minus debt in tens of thousands of dollars and cube rooted). Given that household wealth ranged from −$1,495,000 to $28,000,000, this study used the cube root to adjust for skewness and reduce error variability, consistent with other studies using HRS (Zaborenko et al., 2020). Preliminary analyses found that this transformation did not alter the substantive conclusions.

Further, four adult health-related indicators were included in this study. *Depressive symptoms* have been found to be predictive of the development of disability in later life (Covinsky et al., 2010), thus this study used an 8-item Center for Epidemiological Studies—Depression (CES-D) score in which a higher score indicated more symptoms. *Self-rated health* ranged from 0 (poor) to 4 (excellent). Being physically active has been found to reduce the risk of disability onset and progression (Tak et al., 2013), thus this study used a self-reported *physical activity* scale that gathered both frequency and intensity of exercise. Respondents were asked how often they participated in moderate and vigorous physical activity. A scale was created by weighting the type of physical activity by intensity (moderate = 1.4, vigorous = 1.8) based on metabolic equivalent recommendations, with scores range from 0 (no physical activity) to 12.8 (moderate and vigorous physical activity daily). Finally, *body-mass index* (BMI) was based on self-reports and categorized into underweight or normal weight (BMI < 25), overweight (25 ≥ BMI < 30), and obese (BMI ≥ 30). Although scholars have found that underweight adults have a higher likelihood of experiencing disability (Lingying et al., 2025), this study combined underweight/normal due to a small number of respondents in the underweight category (less than 1%). In preliminary analyses, I also tested for underweight as a separate category but found that it was nonsignificant.

### Analytic Strategy

Analyses were conducted using Stata/SE 18.0. First, *t*-tests and chi square were utilized to examine stress exposure, stress appraisal, and disability across the four racial, ethnic, and nativity groups (Table 1). Second, this study prospectively examined the relationship between chronic stress and disability onset using Weibull accelerated failure time (AFT) models. Most often Weibull AFT models are used by engineers to determine the lifespan of machinery or to predict the warranty of a product (Liu et al., 2023); however, this model is becoming more common in aging and health research (Bauldry et al., 2023; Liu et al., 2023; Sauerteig-Rolston & Ferraro, 2024). One advantage of the Weibull AFT model is the ability to predict time, interpreted as a “time to failure” or “time to onset of X condition.” A Cox model cannot directly predict time; rather Cox models require specifying a certain time frame (e.g., a 15-year study period) and calculating the probability of an event happening during that time (Liu et al., 2023). Weibull AFT models allow for a decreasing, constant, or increasing failure rate over time and for the calculation of a mean or median time to onset. An increasing failure rate (i.e., time to onset) is denoted by a shape parameter (ln_p) that is greater than one, while an ln_p that is less than one reflects a decreasing failure rate over time. In addition, unlike the Cox model, the Weibull AFT model does not rely on a proportional-hazards assumption. In preliminary analyses, I fit a Cox model and found the same pattern of substantive findings, although I also found failures of the proportional-hazards assumption for key independent variables and covariates.

**Table 1.** Descriptive Statistics, Total Sample and by Race, Ethnicity, and Nativity

Variables	Range	Total *N* = 9,171	White *n* = 6,982	Black *n* = 1,310	U.S.-born Hispanic *n* = 392	Foreign-born Hispanic *n* = 487
Incident disability	0.1	0.19	0.18	0.22^a^	0.22^b^	0.21
Age of incident disability^g^	52–102	75.16	77.65	68.25^a^	68.83^b^	68.98^c^
Stress exposure	0–7	2.19	2.09	2.65^a^	2.60^b^	2.08^ef^
Stress appraisal^h^	1–3	1.57	1.59	1.52^a^	1.52^b^	1.59^e^
*Covariates*						
Women	0.1	0.58	0.56	0.65^a^	0.55^d^	0.55^e^
Married	0.1	0.68	0.71	0.49^a^	0.70^d^	0.74^e^
Education (in years)	0–17	13.30	13.66	12.99^a^	12.21^b^^,^^d^	9.93^c^^,^^e^^,^^f^
Household wealth (cube root in $10,000s)	−5.31–14.09	2.75	3.12	1.45^a^	2.07^b^^,^^d^	1.55^c^^,^^f^
Depressive symptoms	0–8	0.96	0.86	1.26^a^	1.35^b^	1.29^c^
Self-rated health	0–4	2.47	2.57	2.17^a^	2.20^b^	2.08^c^
Physical activity	0–12.8	5.39	5.48	4.83^a^	5.39^d^	5.63^e^
BMI						
Underweight/normal (< 25)	0.1	0.29	0.31	0.20^a^	0.19^b^	0.27^c^^,^^e^^,^^f^
Overweight (25 ≥ BMI < 30)	0.1	0.38	0.39	0.35^a^	0.40	0.39
Obese (≥ 30)	0.1	0.33	0.30	0.45^a^	0.41^b^	0.34^c^^,^^e^^,^^f^

*Notes*: Numbers are means or proportions. Significance across subsamples indicated at *p* < .05.

^a^Comparing Black and White adults.

^b^Comparing U.S.-born Hispanic and White adults.

^c^Comparing foreign-born Hispanic and White adults.

^d^Comparing U.S.-born Hispanic and Black adults.

^e^Comparing foreign-born Hispanic and Black adults.

^f^Comparing foreign-born Hispanic and U.S.-born Hispanic adults.

^g^Age of first disability among those respondents who did not experience a disability by W1 but developed one during the study period. (1,760 total respondents; 1,281 White respondents; 290 Black respondents; 88 U.S.-born Hispanic respondents; 101 foreign-born Hispanic respondents).

^h^ *N* of cases is difference across this row because the means are gathered from respondents who experienced at least one ongoing chronic stressor (7,680 total respondents; 5,802 White respondents; 1,146 Black respondents; 334 U.S.-born Hispanic respondents; 398 foreign-born Hispanic respondents).

Similar to other event history methodology, the Weibull AFT model requires two variables: an incidence variable and a duration variable. In this study, the incidence variable is the first report of a disability during the study period, and the age at onset of first disability is the duration variable. Although age of onset of disability is not directly measured, this study utilized respondents birthdate (year and month) and whether a respondent reported a disability at each biennial interview to parameterize the model. Age as the time metric was used to interpret the transition to disability as an age-specific incident function (Thiébaut & Bénichou, 2004). By limiting the sample to those respondents who had no disability prior to or at age reported at W1 (2010 or 2012), I identified those who experienced incident disability (coded 1; 0 = no disability) during the study period (2010/2012–2020). For people who experienced disability, I recorded the age at which they first reported it. For people who did not experience disability, I recorded the last age they were observed in the data (i.e., censored). Results from the event history analysis were interpreted both in terms of the coefficient (β) and time ratio (*e*^β^; Tables 2 and 3). Earlier onset of disability is reflected in a negative β (and *e*^β^ < 1) whereas later onset is reflected in a positive β (and *e*^β^ > 1).

**Table 2.** Weibull Accelerated Failure-Time Models Associated With Stress Exposure and the Incidence of Disability (*N* = 9,171)

Independent variables	Model 1			Model 2			Model 3		
	β	*SE*	Time ratio (*e*^β^)	β	*SE*	Time ratio (*e*^β^)	β	*SE*	Time ratio (*e*^β^)
*Demographics*									
Black^a^	−0.26***	0.02	0.77	−0.22***	0.02	0.81	−0.11***	0.02	0.89
U.S.−born Hispanic^a^	−0.25***	0.04	0.78	−0.22***	0.04	0.80	−0.15***	0.03	0.86
Foreign−born Hispanic^a^	−0.27***	0.03	0.77	−0.26***	0.03	0.77	−0.13***	0.03	0.87
Women^b^	−0.02	0.02	0.98	−0.02	0.02	0.98	−0.01	0.02	0.99
Stress exposure				−0.07***	0.004	0.93	−0.04***	0.005	0.96
*Covariates*									
Married^c^							−0.04*	0.02	0.96
Education							−0.01***	0.003	0.99
Household wealth							0.06***	0.005	1.06
Depressive symptoms							−0.04***	0.004	0.96
Self-rated health							0.08***	0.01	1.08
Physical activity							0.002	0.002	1.00
Overweight^d^							−0.02	0.02	0.98
Obese^d^							−0.16***	0.02	0.86
Constant	3.93***			4.07***			3.88***		
Ln_p	1.12***			1.15***			1.20***		
*Likelihood ratio χ* ^*2*^	188.85			445.93			914.69		

*Notes*: A negative β reflects earlier onset of disability (positive, later onset). *SE* = standard error.

^a^Reference group is White.

^b^Reference group is men.

^c^Reference group is not married.

^d^Reference group is underweight/normal BMI.

^*^ *p* < .05. **p < .01. ***p < .001.

**Table 3.** Weibull Accelerated Failure-Time Models Associated With Stress Appraisal and the Incidence of Disability (*N* = 7,680)

Independent variables	Model 1			Model 2			Model 3		
	β	*SE*	Time ratio (*e*^β^)	β	*SE*	Time ratio (*e*^β^)	β	*SE*	Time ratio (*e*^β^)
*Demographics*									
Black^a^	−0.23***	0.02	0.79	−0.13***	0.02	0.88	0.06	0.07	1.06
U.S.-born Hispanic^a^	−0.21***	0.04	0.81	−0.14***	0.04	0.87	−0.01	0.12	0.99
Foreign-born Hispanic^a^	−0.23***	0.04	0.79	−0.13**	0.04	0.88	−0.44***	0.11	0.65
Women^b^	−0.004	0.02	0.996	−0.004	0.02	0.996	−0.003	0.02	0.997
Stress exposure	−0.07***	0.01	0.93	−0.04***	0.01	0.96	−0.04***	0.01	0.96
Stress appraisal	−0.13***	0.02	0.88	−0.08***	0.02	0.92	−0.07***	0.02	0.93
*Covariates*									
Married^c^				−0.04*	0.02	0.96	−0.04*	0.02	0.96
Education				−0.01***	0.003	0.99	−0.01***	0.003	0.99
Household wealth				0.05***	0.01	1.06	0.05***	0.01	1.06
Depressive symptoms				−0.03***	0.004	0.97	−0.03***	0.005	0.97
Self-rated health				0.07***	0.01	1.08	0.08***	0.01	1.08
Physical activity				0.003	0.002	1.003	0.002	0.002	1.003
Overweight^d^				−0.02	0.02	0.98	−0.02	0.02	0.98
Obese^d^				−0.17***	0.02	0.84	−0.17***	0.02	0.85
*Interactions*									
Stress appraisal × Black							−0.12**	0.04	0.89
Stress appraisal × U.S.-born Hispanic							−0.08	0.07	0.93
Stress appraisal × Foreign-born Hispanic							0.18**	0.06	1.20
Constant	4.27***			4.05***			4.02***		
Ln_p	1.12***			1.17***			1.17***		
*Likelihood ratio χ* ^*2*^	403.79			794.46			815.24		

*Notes*: A negative β reflects earlier onset of disability (positive, later onset). *SE* = standard error.

^a^Reference group is White.

^b^Reference group is men.

^c^Reference group is not married.

^d^Reference group is underweight/normal BMI.

^*^ *p* < .05. **p < .01. ***p < .001.

Third, product terms were added to test whether race, ethnicity, and nativity moderate the relationship between (a) stress exposure and disability and (b) stress appraisal and disability. Finally, sensitivity analyses were conducted to examine the robustness of the findings and are presented in Supplementary Material.

## Results

### Sample Characteristics

Table 1 displays descriptive statistics by race, ethnicity, and nativity. About 19% of the sample experienced an incident disability during the decade of study observation. Black and U.S.-born Hispanic adults were more likely to develop a disability than White adults. The average age of disability onset among respondents who developed a disability during the study period was about 75 years; however, Black, U.S.-born Hispanic, and foreign-born Hispanic adults developed disability at younger ages than White adults. For example, the average age of incident disability for Black adults was about 68 years of age, but about 78 years for White adults.

Among all respondents, the average chronic stress exposure was 2.19 (out of 7), with Black and U.S.-born Hispanic adults reporting significantly more stress than White and foreign-born Hispanic adults (respectively, 2.65, 2.60, 2.09, 2.08). Although Black adults reported higher levels of exposure to chronic stress than White adults, among those that reported at least one exposure, the average stress appraisal was higher (i.e., more negative/upset) for White adults (1.59) and foreign-born Hispanic adults (1.59) than Black adults (1.52). See Supplementary Table 1 for the frequency distribution of the cumulative measure of exposure and appraisal and each indicator across racial, ethnic, and nativity groups.

### Stress Exposure and Disability Incidence

To assess the influence of chronic stress exposure on incident disability, estimates from Weibull accelerated failure-time models are presented in Table 2. Model 1 included demographics only and revealed that Black, U.S.-born Hispanic, and foreign-born Hispanic adults were more likely to experience the onset of disability at a younger age than White adults (respectively, β = −0.26, β = −0.25, β = −0.27, each *p *< .001). The negative slope revealed that the age of disability onset was earlier for these groups, and the time ratios (respectively, *e*^β^ = 0.77, *e*^β^ = 0.78, *e*^β^ = 0.77) mean that Black, U.S.-born Hispanic, and foreign-born Hispanic adults experienced disability in roughly 77% of the time as White adults (among those that were disability-free at W1). Model 2 included stress exposure and revealed that persons reporting higher levels of exposure were more likely to experience an earlier onset of disability (β = −0.07, *p* < .001). The negative coefficient for a continuous variable revealed that time to disability onset decreased by a factor of 0.93 (exp(−0.07)) for each additional stress exposure. Stress exposure remained associated with an earlier onset of disability after adjusting for covariates in Model 3 (β = −0.04, *p* < .001). Although slightly attenuated, the likelihood of disability remained greater for Black, U.S.-born Hispanic, and foreign-born Hispanic adults than White adults. Black, U.S.-born Hispanic, and foreign-born Hispanic adults experienced a 10% [(1−*e*^−0.11^) × 100], 14%, and 13% earlier time to onset of disability compared with White adults, respectively. However, the relationship between stress exposure and disability onset did not vary by race, ethnicity, and nativity.

### Stress Appraisal and Disability Incidence

Table 3 presents WAFT models accounting for both chronic stress exposure and appraisal. Model 1 included demographic factors, stress exposure, and stress appraisal. After adjusting for stress exposure, more upsetting levels of appraisal were associated with an earlier onset of disability (β= −0.13, *p* < .001). Although slightly attenuated, exposure, and appraisal remained significant in Model 2 after adjusting for covariates. Model 3 displays the interaction between stress appraisal and race, ethnicity, and nativity. This model revealed that stress appraised as more upsetting had a more detrimental influence on later-life disability for Black adults than White adults (β = −0.12, *p < *.01), reflected in a roughly 11% [(1−*e*^*−0.12*^) × 100] earlier time to onset. In contrast, appraising stress as more upsetting had a protective influence on later-life disability for foreign-born Hispanic adults compared with White adults (β = 0.18, *p < *0.01), reflected in about a 20% [(1−*e*^*0.18*^) × 100] later time to onset, after adjusting for total number of chronic stressors.

**Figure 1.**
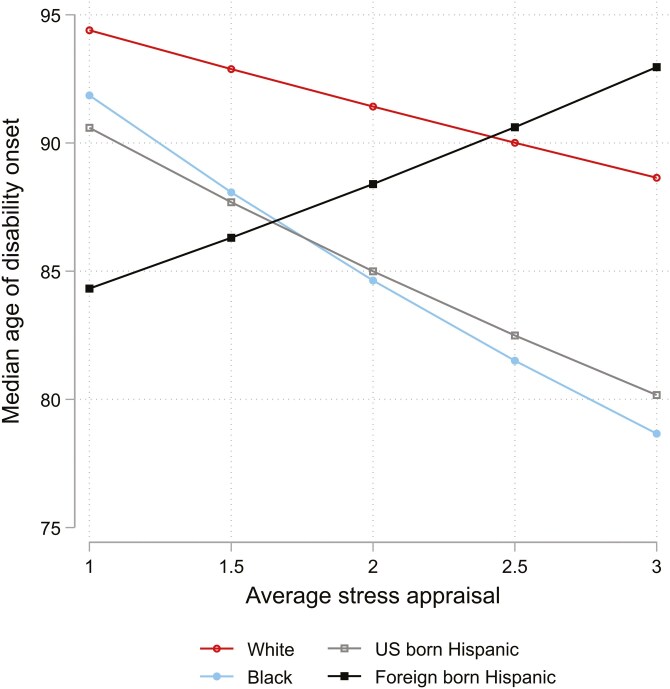
Predicted Median Age of Disability Onset by Average Stress Appraisal (Among Respondents with At Least One Exposure) and Race, Ethnicity, and Nativity.

To further examine these instances of statistical moderation, Figure 1 illustrates that Black adults who reported an average stress appraisal score of 3 had a median age of disability onset that was about 10 years earlier than White adults reporting the same stress appraisal score. The relationship between stress appraisal and the median age of disability onset for US-born Hispanic adults was similar to Black adults, although not statistically significantly different from White adults. At lower levels of an upsetting appraisal, foreign-born Hispanic adults had a significantly earlier median age of disability onset than White adults, but at higher levels of appraisal, foreign-born Hispanic adults trend towards a later age of disability onset. This crossover occurred around an average appraisal score of 2.4.

## Discussion and Implications

The present study assessed disparities in chronic stress and disability among adults in later life. Most research examining stress and health has examined one component of stress—exposure—but people who are exposed to the same stressor may experience distinct outcomes, in part due to the corresponding appraisal of the exposure. Many adults are eager to maintain independence and physical functioning, yet this study showed that both high levels of chronic stress exposure and negative appraisals were an impediment to achieving this goal. Results revealed that chronic stress exposure was detrimental for the onset of later-life disability, but in some cases racial, ethnic, and nativity disparities in disability may be further exacerbated by more upsetting appraisals of stress.

### Chronic Stress and Disability

Addressing the first research question, this study found significant differences in chronic stress exposure, appraisal, and disability in later life by race, ethnicity, and nativity. Results from the descriptive analysis revealed that Black and U.S.-born Hispanic adults experienced greater chronic stress exposure than White and foreign-born Hispanic adults. In fact, Black adults had a higher likelihood of experiencing six out of the seven exposures within the overall chronic stress exposure measure and U.S.-born Hispanic adults had a higher likelihood of experiencing four out of the seven exposures compared with White adults.

Among those who reported an affirmative response to at least one stressor, White and foreign-born Hispanic adults on average reported their stress exposure as more upsetting than Black adults. This finding is consistent with prior studies examining racial, ethnic, and nativity disparities in chronic stress appraisal (e.g., Brown et al., 2020a). It is unclear why Black adults reported higher levels of exposure and lower levels of appraisal, but I present two related explanations.

First, the stress process theory posits that stress is “rooted in conditions of life signified by social statuses and roles” (Wheaton 2009, p. 236). Perhaps Black adults, who are exposed to high levels of stress throughout the life course, are better able to manage their psychological and emotional reactions to these chronic stressors. Brown et al., (2020a) argued that Black adults may not “conform” to the stereotypical reaction to stress, but rather have learned to develop alternative interpretations and meanings for stress exposure. For example, they may engage in a compensatory coping process which argues that if there is persistent stress in a role, an individual may devalue the importance of that role and seek out new roles that may be beneficial (Thoits, 2009).

Second, the stress inoculation model suggests that a history of stress buffers against subsequent stress, creating a “steeling” effect (Rudolph & Flynn, 2007). In other words, exposure to a broad range of stressors may reduce the effects of subsequent events (Turner, 2009). Prior research has found that Black adults experience more exposure to stressors across the life course (Boen, 2020; Sauerteig-Rolston & Ferraro, 2024; Williams et al., 2019); thus, Black adults may have developed resilience to the emotional response of these difficult life circumstances.

Consistent with previous literature, Black, and U.S.-born Hispanic adults were at higher risk of developing a disability during the study period than White adults (e.g., Boen & Hummer, 2019; Garcia et al., 2017; Sauerteig-Rolston & Ferraro, 2024). Moreover, the onset was accelerated (i.e., occurred at an earlier age) for Black, U.S.-born Hispanic, and foreign-born Hispanic adults compared with White adults.

### The Relationship Between Chronic Stress and Disability

Addressing the second research question revealed that chronic stressors were associated with worse physical functioning, consistent with the stress process theory. Both higher levels of chronic stress exposure and more upsetting levels of stress appraisal were associated with earlier onset of a disability throughout the study period. This finding is consistent with others that explored aspects of emotional reactivity to stress and physical functioning (Leger et al., 2018; Rush et al. 2025) and addresses an understudied component of stress process theory, revealing that emotional reactivity is perhaps more detrimental for physical functioning than the exposure itself.

One potential mechanism that may explain the association between stress appraisal and disability is engagement in negative health behaviors as a coping mechanism. Scholars report that health behaviors are largely influenced by stress levels because they can be used to “cope with or manage the distress they experience” (Park & Iacocca, 2014; p. 124). Although some literature suggests that negative emotional reactivity may lead to health promoting behavior such as higher levels of exercise, others have found that negative feelings are associated with health destructive behaviors such as higher levels of fast-food intake, increased alcohol consumption, less exercise, and more smoking (Park & Iacocca, 2014). Engaging in negative health behaviors may be associated with more negative appraisal of stressful experiences and thus perpetuate difficulties in physical functioning in later life.

### Disparities in the Relationship Between Chronic Stress Appraisal and Disability

Addressing the third research question revealed distinct relationships among stress, disability, and race, ethnicity, and nativity. This study did not find evidence that race, ethnicity, or nativity moderated the relationship between exposure and disability. Yet, there were intriguing findings related to differences in the implications of appraisal on disability for Black and foreign-born Hispanic adults compared with White adults. At lower levels of stress appraisal (i.e., experiencing a stressor but not being upset) there was not a significant difference in age of onset of disability among White and Black adults; however, at higher levels (i.e., reporting being somewhat or very upset by stress), Black adults experienced the onset of disability at a significantly earlier age than White adults.

I offer two potential explanations for why Black adults were especially hard hit by negative stress appraisal. First, descriptive statistics revealed that Black adults that experienced financial strain were more likely to be upset by the exposure than White adults. Historical and contemporary racism found in all institutions within the United States has drastically influenced the socioeconomic status of Black Americans (Phelan & Link, 2015). Financial strain and other SES outcomes are “severe and often unrelenting stressors patterned by social and structural disadvantage over the life course” (Brown et al., 2020b, p. 10). Perhaps being upset by these stressors is more detrimental for Black adults because they want to climb the social ladder and improve their financial and housing situation, but they live in a racialized social structure that continues to make each step more difficult and inhibits the control they have over social mobility.

I conducted a sensitivity analysis including a sum indicator for perceived constraints on personal control, described in Supplementary Material, but the findings did not alter the substantive conclusions. These indicators did not directly relate to personal control regarding specific chronic stressors but rather life situations in general (e.g., “I have little control over the things that happen to me,” “I often feel helpless in dealing with the problems of life. . .”) Future research should examine if perceived control related specifically to chronic stressors alters the influence of appraisal on health outcomes.

Second, White adults may have more resources to mitigate the harmful effect of negative reactivity to stress, whereas Black adults may lack these resources. Indeed, the differential vulnerability hypothesis argues that socially disadvantaged groups have more negative outcomes to stress because they have fewer resources to buffer the noxious effects (Brown et al., 2020b; Kessler, 1979). In this study, Black adults fared worse both in terms of SES resources (education and household wealth) and health-related factors (depressive symptoms, self-rated health, obesity, and physical activity). As such, to mitigate the deleterious effect of negative stress appraisal for Black adults, it is important to address the historical and persistent racism present in key social institutions in the United States that limit the number of resources available.

Among foreign-born Hispanic adults who experienced at least one chronic stress exposure, appraising the exposure(s) as upsetting was protective against disability onset. Indeed, foreign-born Hispanic adults who reported being very upset by stress had a median age of disability onset roughly four years later than White adults with the same level of appraisal. However, caution is warranted in interpreting this trend for two reasons. First, when examining foreign-born Hispanic adults only, this study did not find an association between stress appraisal and disability. Second, only about 8% of both foreign-born Hispanic and White respondents experiencing chronic stress reported an average stress appraisal score at or above 2.5. Examining confidence intervals around the data, I found that the confidence intervals did not overlap (meaning a significant difference) only at a lower level of appraisal. Foreign-born Hispanic adults had the earliest age of disability onset when they reported experiencing at least one chronic stressor, but not being upset. In fact, foreign-born Hispanic adults with an average stress appraisal score of one experienced the onset of disability about 10 years earlier than White adults. Despite this, a sensitivity analysis in which I created a high versus low-stress appraisal indicator, revealed that feeling upset by stress exposure was protective for foreign-born Hispanic adults compared with White adults. This finding is particularly intriguing and requires further exploration, but I provide one potential explanation for this trend.

One body of literature argues that negative stress appraisal generates the body’s stress response system, which often has undesirable consequences (Lazarus & Folkman, 1984; Morris et al., 2022). However, it is also plausible that negative appraisal may not be invariably adverse. Scholars have found that foreign-born Hispanic adults report higher levels of social support, specifically from family, than white adults (Almeida et al., 2009). Perhaps being very upset by chronic stressors leads an individual to seek help or triggers a response from social network members to provide or find methods of support to overcome disadvantaged life circumstances. Among foreign-born Hispanic adults, studies have found that acculturation factors such as language barriers and different cultural values and religious beliefs decrease help-seeking behavior (Ishikawa et al., 2010). However, showing signs of distress may prompt others to step in and provide support even if someone is unsure how to ask for that help, which over the long haul may be protective for physical functioning. In contrast, going on as if nothing happened and suppressing feelings may have negative implications for disability for foreign-born Hispanic adults (Harrell, 2000).

### Limitations and Future Directions

Although this study deepens our understanding of disparities in chronic stress and health by assessing appraisal of exposure, there are several limitations. First, this study utilized a measure of appraisal specified in other studies, but as Brown et al., (2020b) note, these measures ask whether the exposure occurred for 12 months or longer. Questions regarding chronic stress are presented to HRS respondents every 4 years, thus it is possible that respondents are not *currently* experiencing the chronic stressor, and are providing retrospective stress responses, which may be biased. Second, accounting for appraisal revealed unique relationships between stress, disability, and race, ethnicity, and nativity; however, a more thorough analysis of understanding the meaning people place behind their appraisal is warranted. For example, what factors cause someone to feel like an exposure is upsetting or not? This line of research would benefit from a qualitative analysis, diving into more details on the subjectivity of stress exposure. Third, this study focused on the cumulative burden of chronic stress exposure and appraisal; however, it may be beneficial for future research to disentangle which stressors are more detrimental for different groups of people.

These findings have important practical and policy implications. It would be beneficial for health care institutions, public programs, and social workers to assess the emotional reactivity to stress exposure before and after providing an intervention. Perhaps specific resources such as support groups for people facing relationship strain, financial planning, resources for people dealing with financial and housing difficulties, and respite services to lighten caregiver burden could reduce negative appraisal to enduring stressors. Paying close attention to specific cultural factors and network interpretations may clarify how diverse populations respond to stress and ultimately reduce health disparities in later life.

## Conclusion

This study emphasized the influence of chronic stress exposure and appraisal on later-life disability among White, Black, U.S.-born Hispanic, and foreign-born Hispanic older adults. I did not find a difference between exposure and disability by race, ethnicity, and nativity, but the separation of exposure and appraisal revealed unique differences across groups—and those differences have long lasting effects on disability in later life. Although Black adults experienced higher stress exposure, they reported being less upset. Despite this, Black adults who were more upset by stress exposure had an earlier onset of disability than White adults. In addition, foreign-born Hispanic adults who reported being less upset by chronic stressors experienced an earlier onset of disability than White adults, but this trend flipped among foreign-born Hispanic adults who reported being more upset by stress exposure. Stress exposure does not influence everyone in the same way, and the subjective meaning of stress is imperative for understanding the relationship between stress and health in later life. Reducing disparities in disability during later life will require resources to ameliorate not just the exposure to stress, but also to alleviate the negative emotions that come with chronic disadvantages or find constructive ways to cope with the stressors that are judged to be upsetting.

## Supplementary Material

igaf024_suppl_Supplementary_Table_S1

## Data Availability

All data files for this analysis are available from the University of Michigan and the Health and Retirement Study. Data can be obtained from the HRS website: https://hrs.isr.umich.edu/data-products. The study reported in the manuscript was not preregistered.
