# Supplementary material for: Disparities in Chronic Stress Exposure and Appraisal and Later-Life Disability
Source: Innov Aging. 2025 Apr 4;9(5):igaf024. doi: 10.1093/geroni/igaf024 (PMC12123066; doi:10.1093/geroni/igaf024)
Supplement: igaf024_suppl_Supplementary_Table_S1 [file igaf024_suppl_supplementary_table_s1.docx]

***Innovation in Aging* Supplementary Material:** Sauerteig-Rolston. **Disparities in Chronic Stress Exposure and Appraisal and Later-Life Disability.**

*Sensitivity Analyses*

Several sensitivity analyses were conducted to examine the robustness of our results. I conducted three analyses utilizing different approaches for the conceptualization of stress appraisal. First, instead of calculating the average appraisal score among endorsed chronic stressors only, I calculated the average of the exposure and appraisal combined variable (range 0-3). In this analysis (N=9,171), I found that the interaction between race, ethnicity, and nativity X stress appraisal was not significant among foreign-born Hispanic respondents (p=0.054).

Second, I created a sum score, (range 0-21; in which a 0 represents a response of “no, did not happen” to all 7 stressors and 21 represents a response of “yes, very upsetting” to all 7 stressors. In this analysis (N=8,754; had to be non-missing on stress exposure), I had consistent findings in terms of the race, ethnicity, and nativity X stress appraisal interaction. In both sensitivity checks, I found that (1) US-born Hispanic adults had an earlier onset of disability than White adults, and (2) stress exposure was non-significant. This may seem like an interesting finding; however, I argue that these two approaches combine exposure and appraisal scores into one indicator, making it more difficult to decipher if it’s the exposure or the appraisal that has a bigger influence on disability.

Third, because relatively few respondents reported high levels of stress appraisal, a high versus low stress appraisal indicator was created (i.e., above and below the mean level of feeling upset; 1.572) and similar patterns in terms of the interaction effect were found, with higher levels of appraisal being disadvantageous for Black adults compared to White adults (β = -0.08 p = 0.062), but protective for foreign-born Hispanic adults (β = 0.15, p < 0.05).

In addition, I conducted two analyses aimed at understanding selection and mortality. I utilized logistic regression to examine the relationship between stress exposure and appraisal and whether respondents had a disability at the initial survey (Supplementary Table 2). This analysis was informative for understanding the occurrence of disability and for identifying the context and potential selection associated with the longitudinal analysis. Respondents who reported higher levels of stress exposure and appraisal had higher odds of disability at W1. More negative health-related factors (being overweight or obese [compared to normal weight], higher levels of depressive symptoms, lower levels of physical activity, and poorer self-rated health) and lower SES resources (education and wealth) had higher odds of disability at W1 and thus were more likely to be excluded from the longitudinal analysis. There were no differences among race, ethnicity, nativity, and gender among respondents who reported a disability at W1 compared to those who did not. Also, although I incorporated information from respondents who died by 2020 into the analysis, I tested whether the conclusions might be due to selective survival using Heckman’s adjustment (Heckman 1979). I found that the conclusions were unchanged.

Other sensitivity analyses included: (1) excluding “ongoing health problems” in the stress exposure and appraisal indicators because this could reflect some difficulty with physical functioning; the main conclusions were sustained; (2) testing whether factors related to perceived control influenced disparities in the relationship between appraisal and disability, but the findings presented herein remained, and (3) stratifying the Weibull AFT models by race, ethnicity, and nativity and findings are presented in Supplementary Table 3. In the model examining foreign-born Hispanic respondents only, I tested whether there were differential effects of stress appraisal on disability onset by (1) interview language (English or Spanish), and (2) age at immigration (18 years and younger or older than 18 years, i.e., spent childhood outside of the US) and found no differences.

| Supplementary Table 1. Frequency distribution for stress exposure and appraisal indicators by race, ethnicity, and nativity | | | | | | | | | | | | |
| --- | --- | --- | --- | --- | --- | --- | --- | --- | --- | --- | --- | --- |
|  |  | | Total  9171 | White  6,982 | | Black  1,310 | | US born- Hispanic  392 | | Foreign-born Hispanic  487 | |  |
| EXPOSURE TO CHRONIC STRESSOR | | | | | | | | | | | | |
| Health problems (in oneself) | 0,1 | | 0.60 | 0.59 | | 0.63^a^ | | 0.62 | | 0.58^e^ | |  |
| Physical or emotional problems (in family) | 0,1 | | 0.37 | 0.36 | | 0.35 | | 0.41^d^ | | 0.38 | |  |
| Alcohol or drug use (in family) | 0,1 | | 0.15 | 0.14 | | 0.17^a^ | | 0.20^b^ | | 0.11^cef^ | |  |
| Financial strain | 0,1 | | 0.42 | 0.38 | | 0.61^a^ | | 0.55^bd^ | | 0.45^cef^ | |  |
| Housing problems | 0,1 | | 0.14 | 0.10 | | 0.29^a^ | | 0.23^bd^ | | 0.16^cef^ | |  |
| Relationship problems | 0,1 | | 0.21 | 0.20 | | 0.27^a^ | | 0.28^b^ | | 0.17^ef^ | |  |
| Caregiving | 0,1 | | 0.33 | 0.32 | | 0.36^a^ | | 0.34 | | 0.26^cef^ | |  |
| APPRAISAL OF CHRONIC STRESSOR (among those that did experience the stressor) | | | | | | | | | | | | |
| Health problems (in oneself) (N=5462) |  |  | |  |  | |  | |  | |  |  |
| Yes happened, but not upsetting | 0,1 | 0.53 | | 0.52 | 0.57^a^ | | 0.56 | | 0.48^e^ | |  |  |
| Yes happened, somewhat upsetting | 0,1 | 0.39 | | 0.39 | 0.35^a^ | | 0.33^b^ | | 0.44^f^ | |  |  |
| Yes happened, very upsetting | 0,1 | 0.09 | | 0.09 | 0.07 | | 0.11 | | 0.09 | |  |  |
| Physical or emotional problems (in family) (N=3269) |  |  | |  |  | |  | |  | |  |  |
| Yes happened, but not upsetting | 0,1 | 0.36 | | 0.34 | 0.43^a^ | | 0.47^b^ | | 0.38 | |  |  |
| Yes happened, somewhat upsetting | 0,1 | 0.48 | | 0.49 | 0.44^a^ | | 0.36^b^ | | 0.52^f^ | |  |  |
| Yes happened, very upsetting | 0,1 | 0.17 | | 0.18 | 0.13^a^ | | 0.16 | | 0.10^c^ | |  |  |
| Alcohol or drug use (in family) (N=1317) |  |  | |  |  | |  | |  | |  |  |
| Yes happened, but not upsetting | 0,1 | 0.28 | | 0.27 | 0.36^a^ | | 0.35 | | 0.24 | |  |  |
| Yes happened, somewhat upsetting | 0,1 | 0.48 | | 0.49 | 0.47 | | 0.38 | | 0.51 | |  |  |
| Yes happened, very upsetting | 0,1 | 0.24 | | 0.25 | 0.17^a^ | | 0.27 | | 0.25 | |  |  |
| Financial strain (N=3859) |  |  | |  |  | |  | |  | |  |  |
| Yes happened, but not upsetting | 0,1 | 0.49 | | 0.51 | 0.46^a^ | | 0.49 | | 0.37^cef^ | |  |  |
| Yes happened, somewhat upsetting | 0,1 | 0.38 | | 0.37 | 0.39 | | 0.39 | | 0.47^cf^ | |  |  |
| Yes happened, very upsetting | 0,1 | 0.13 | | 0.13 | 0.15 | | 0.11 | | 0.17 | |  |  |
| Housing problems (N=1255) |  |  | |  |  | |  | |  | |  |  |
| Yes happened, but not upsetting | 0,1 | 0.57 | | 0.58 | 0.56 | | 0.62 | | 0.40^cef^ | |  |  |
| Yes happened, somewhat upsetting | 0,1 | 0.33 | | 0.32 | 0.34 | | 0.29 | | 0.44^cf^ | |  |  |
| Yes happened, very upsetting | 0,1 | 0.10 | | 0.10 | 0.09 | | 0.09 | | 0.16^cef^ | |  |  |
| Relationship problems (N=1925) |  |  | |  |  | |  | |  | |  |  |
| Yes happened, but not upsetting | 0,1 | 0.47 | | 0.43 | 0.55^a^ | | 0.57^b^ | | 0.57^c^ | |  |  |
| Yes happened, somewhat upsetting | 0,1 | 0.41 | | 0.44 | 0.36^a^ | | 0.29^b^ | | 0.33^c^ | |  |  |
| Yes happened, very upsetting | 0,1 | 0.12 | | 0.13 | 0.10 | | 0.14 | | 0.11 | |  |  |
| Caregiving (N=2961) |  |  | |  |  | |  | |  | |  |  |
| Yes happened, but not upsetting | 0,1 | 0.66 | | 0.64 | 0.69^a^ | | 0.74^b^ | | 0.76^c^ | |  |  |
| Yes happened, somewhat upsetting | 0,1 | 0.26 | | 0.27 | 0.25 | | 0.20 | | 0.15^ce^ | |  |  |
| Yes happened, very upsetting | 0,1 | 0.09 | | 0.09 | 0.06 | | 0.06 | | 0.09 | |  |  |
| ^a^ Comparing Black and White adults.  ^b^ Comparing US-born Hispanic and White adults.  ^c^ Comparing foreign-born Hispanic and White adults.  ^d^ Comparing US-born Hispanic and Black adults.  ^e^ Comparing foreign-born Hispanic and Black adults.  ^f^ Comparing foreign-born Hispanic and US-born Hispanic adults. | | | | | | | | | | | | |

| **Supplementary Table 2.** Binary Logistic Regression Model Associated with Chronic Stressors and Disability Occurrence at or before Wave 1 (N=10,852) | | |
| --- | --- | --- |
|  | OR | SE |
| *Demographics* |  |  |
| Black^a^ | 1.16* | 0.08 |
| US-born Hispanic^a^ | 1.05 | 0.13 |
| Foreign-born Hispanic^a^ | 1.18 | 0.13 |
| Age at W1 | 1.06*** | 0.003 |
| Women^b^ | 0.99 | 0.05 |
| Stress exposure | 1.13*** | 0.02 |
| Stress appraisal | 1.35*** | 0.06 |
| *Covariates* |  |  |
| Married | 0.98 | 0.05 |
| Education | 0.96*** | 0.01 |
| Household wealth | 0.95** | 0.02 |
| Depressive symptoms | 1.19*** | 0.02 |
| Self-rated health | 0.55*** | 0.02 |
| Physical activity | 0.93*** | 0.01 |
| Overweight^c^ | 1.20** | 0.08 |
| Obese^c^ | 1.64*** | 0.11 |
| Notes: OR=odds ratio; SE=standard error  ^a^Reference group is White.  ^b^Reference group is men.  ^c^Reference group is underweight/normal  **p* < .05; ***p* < 0.01; ****p* < .001. | | |

| **Supplementary Table 3.** Weibull accelerated failure-time models associated with stress appraisal and the incidence of disability, stratified by race, ethnicity, and nativity | | | | | | | | | | | | | | | |  |
| --- | --- | --- | --- | --- | --- | --- | --- | --- | --- | --- | --- | --- | --- | --- | --- | --- |
|  | White  (n=5,802) | | | | Black  (n= 1,146) | | | | US-born Hispanic (n=334) | | | | Foreign-born Hispanic  (n=398) | | |  |
|  | β | SE | Time ratio  (*e*^β^) | β | | SE | Time ratio  (*e*^β^) | β | | SE | Time ratio  (*e*^β^) | β | | SE | Time ratio  (*e*^β^) | |
| *Demographics* |  |  |  |  | |  |  |  | |  |  |  | |  |  | |
| Women^a^ | 0.002 | 0.02 | 1.002 | -0.02 | | 0.06 | 0.98 | 0.05 | | 0.10 | 1.05 | -0.23* | | 0.10 | 0.79 | |
| Stress exposure | -0.04*** | 0.01 | 0.96 | -0.06*** | | 0.02 | 0.94 | -0.05 | | 0.03 | 0.95 | -0.06* | | 0.03 | 0.94 | |
| Stress appraisal | -0.08*** | 0.02 | 0.93 | -0.19*** | | 0.05 | 0.83 | -0.11 | | 0.09 | 0.90 | 0.15 | | 0.10 | 1.17 | |
| *Adult resources* |  |  |  |  | |  |  |  | |  |  |  | |  |  | |
| Married^b^ | -0.04* | 0.02 | 0.96 | -0.01 | | 0.05 | 0.995 | -0.12 | | 0.11 | 0.89 | -0.24* | | 0.11 | 0.79 | |
| Education | -0.01* | 0.004 | 0.99 | -0.02* | | 0.01 | 0.98 | -0.04* | | 0.02 | 0.96 | 0.000 | | 0.01 | 1.000 | |
| Household wealth | 0.04*** | 0.01 | 1.05 | 0.08*** | | 0.02 | 1.09 | 0.11*** | | 0.03 | 1.11 | 0.06* | | 0.03 | 1.07 | |
| *Adult health* |  |  |  |  | |  |  |  | |  |  |  | |  |  | |
| Depressive symptoms | -0.02*** | 0.005 | 0.98 | -0.05*** | | 0.01 | 0.95 | -0.09*** | | 0.02 | 0.92 | -0.06** | | 0.02 | 0.94 | |
| Self-rated health | 0.06*** | 0.01 | 1.07 | 0.10** | | 0.03 | 1.11 | 0.12* | | 0.05 | 1.13 | 0.08 | | 0.06 | 1.09 | |
| Physical activity | 0.001 | 0.002 | 1.001 | 0.002 | | 0.01 | 1.002 | 0.02 | | 0.01 | 1.02 | 0.03 | | 0.01 | 1.03 | |
| Overweight^c^ | -0.03 | 0.02 | 0.97 | 0.16* | | 0.07 | 1.17 | -0.19 | | 0.14 | 0.83 | -0.21 | | 0.12 | 0.81 | |
| Obese^c^ | -0.17*** | 0.02 | 0.85 | -0.13* | | 0.07 | 0.88 | -0.30* | | 0.13 | 0.74 | -0.18 | | 0.12 | 0.84 | |
| Constant | 3.97*** |  |  | 4.26*** | |  |  | 4.38*** | |  |  | 3.75*** | |  |  | |
| Ln_p | 1.32*** |  |  | 0.89*** | |  |  | 0.98*** | |  |  | 0.93*** | |  |  | |
| *Likelihood Ratio χ^2^* | 438.43 |  |  | 154.85 | |  |  | 71.76 | |  |  | 41.23 | |  | | |
| Notes: A negative β reflects earlier onset of disability (positive, later onset). SE = standard error.  ^a^Reference group is men. ^b^Reference group is not married. ^c^Reference group is underweight/normal BMI.  **p* < .05; ***p* < 0.01; ****p* < .001. | | | | | | | | | | | | | | | |  |

References

Heckman, J. J. (1979). Sample selection bias as a specification error. *Econometrica*, 47(1), 153-161. <https://doi.org/10.2307/1912352>
